# Caregivers' Intention to Vaccinate Their Children Under 12 Years of Age Against COVID-19: A Cross-Sectional Multi-Center Study in Milan, Italy

**DOI:** 10.3389/fped.2022.834363

**Published:** 2022-05-30

**Authors:** Maurizio Lecce, Gregorio Paolo Milani, Carlo Agostoni, Enza D'Auria, Giuseppe Banderali, Giacomo Biganzoli, Luca Castellazzi, Costanza Paramithiotti, Elisabetta Salvatici, Paola Tommasi, Gian Vincenzo Zuccotti, Paola Marchisio, Silvana Castaldi

**Affiliations:** ^1^Department of Biomedical Sciences for Health, Postgraduate School of Public Health, University of Milan, Milan, Italy; ^2^Department of Clinical Science and Community Health, University of Milan, Milan, Italy; ^3^Fondazione IRCCS Ca' Granda Ospedale Maggiore Policlinico, Milan, Italy; ^4^Department of Pediatrics, Ospedale dei Bambini Vittore Buzzi, University of Milan, Milan, Italy; ^5^Department of Pediatrics, AO San Paolo, ASST Santi Paolo e Carlo, University of Milan, Milan, Italy; ^6^Unit of Medical Statistics, Biometry and Epidemiology, Department of Biomedical and Clinical Sciences (DIBIC) “L. Sacco” & DSRC, Luigi Sacco University Hospital, Milan, Italy; ^7^Department of Biomedical and Clinical Sciences – L. Sacco, University of Milan, Milan, Italy; ^8^Department of Pathophysiology and Transplantation, University of Milan, Milan, Italy

**Keywords:** SARS-CoV-2, vaccine hesitancy, infants, children, adolescents, vaccination, parents, caregivers

## Abstract

The impact of Coronavirus disease 2019 (COVID-19) on the pediatric population is increasingly recognized. A widespread vaccination in childhood would provide benefits for children and might help ending the pandemic by enhancing community protection. Following recent approval by the European Medicines Agency (EMA) of Comirnaty (Pfizer-BioNTech) for children aged 5–11 years, we aimed to investigate caregivers' intention to vaccinate their children <12 years of age against COVID-19. A structured questionnaire was administered to caregivers of children aged <12 years visiting the Emergency Department or the outpatient clinics in three major hospitals of Milan, Italy, from 20 September to 17 October 2021. A total of 612 caregivers were invited to participate and 604 accepted (response rate >98%). Three questionnaires were excluded due to compiling errors and 601 were included in the analysis. A total of 311 (51.7%) caregivers stated they would have their child vaccinated, 138 (23%) would refuse to vaccinate their child and 152 (25.3%) were unsure. The intention to vaccinate the child was higher in caregivers vaccinated against COVID-19, in those with a bachelor's degree or higher level of education, and in those with friends/acquaintances who became ill or died due to COVID-19. This study shows that increasing efforts are necessary to provide evidence-based tailored information to caregivers and to promote vaccination in this pediatric age group.

## Introduction

Coronavirus disease 2019 (COVID-19) has been exerting an enormous impact on pediatric population (1). Although COVID-19 is often mild or even asymptomatic in childhood (2), some affected children may experience serious conditions such as pneumonia, the Multisystem Inflammatory Syndrome associated with COVID-19 (MIS-C) (3) and long COVID (4). In Italy, by the end of November 2021, more than 800.000 children have been infected by Severe Acute Respiratory Syndrome Coronavirus 2 (SARS-CoV-2). Among them, more than 8,500 required hospitalizations, 251 were admitted to an intensive care unit (ICU) and 35 died (5). Furthermore, the persistent threat of school closing, home online teaching, quarantine, and social isolation measures had undoubted consequences on children's physical and psychological well-being (6–8).

While two COVID-19 vaccines – Comirnaty by Pfizer-BioNTech and Spikevax by Moderna – were previously authorized in the European Union for individuals aged 12 years and over, on 25 November 2021 the European Medicines Agency (EMA) authorized the use of Comirnaty in children aged 5–11 years (9). The importance of extending COVID-19 vaccination to children aged <12 years was also highlighted by data from the Italian Health Institute showing that COVID-19 cases in Italy started to rise again in November 2021 and that the 6–11-years-old age group held the greatest incidence rates 5. The administration of a COVID-19 vaccine in children and adolescents might also be helpful to limit the spread of SARS-COV-2 by reinforcing community protection and eventually – though this topic is still debated – by achieving herd immunity (10, 11).

Vaccine hesitancy is considered by WHO as one of the ten major threats to global health (12) and it is defined as the “delay in acceptance or refusal of vaccination despite availability of vaccination services. influenced by factors such as complacency, convenience, and confidence” (7). The (lack of) confidence might regard mistrust in the system delivering the vaccine (pharmaceutical companies, vaccination programs decision makers, healthcare providers etc.), the safety and/or the effectiveness of the vaccine. The “no-vax” movement refers properly to this dimension, since most “no vax” individuals do not consider vaccination from an analytical viewpoint but, in many cases, reject it due to a strongly negative attitude toward a “conspiring” system (policy decision makers, pharmaceutical companies etc.), and/or misconceptions about vaccine safety or effectiveness (13). The complacency dimension refers to people who perceive as low the risks related to the disease (risk of getting the disease, risk to develop a severe disease), so they do not consider vaccination as necessary (14). Convenience concerns are expressed by individuals who delay/refuse vaccination because they do not find it appealing, firstly in terms of easy accessibility (14). Calculation was proposed by C. Betsch et al. as a fourth factor and it was described as an extensive information search of pros and cons of a given vaccination with the selfish aim to maximize the utility for oneself, leading to refuse vaccination if risks related to the disease seem to be lower than those related to vaccination (15). Together, confidence, complacency, convenience, and calculation constitute the “4 Cs” model of vaccine hesitancy.

Since the parents'/caregivers' willingness to have their children vaccinated is a *condicio sine qua non* for the success of the vaccination campaign, understanding the reasons for parental vaccine hesitancy is essential for promoting effective vaccination programs.

In Western countries a long-standing tradition of vaccine hesitancy is found in adults, both for themselves (e.g., influenza vaccine, tetanus-diphtheria-pertussis vaccine etc.) (16–19) and their offsprings (e.g., routine childhood vaccines, HPV vaccine etc.) (20, 21). In Italy, the prevalence of parental vaccine hesitancy has been estimated to be 35% (22). Concerning COVID-19 vaccination after initial hesitancy shown by the public (23) and (even more surprisingly) by healthcare workers (24), a sustained adherence to vaccination programs was registered in the Italian adult population (25) and as of November 2021 84.7% of people aged ≥12 years have completed their primary vaccination course (26). However, the beliefs and attitudes of Italian parents/caregivers regarding COVID-19 vaccination of children under 12 years of age remain unclear and of great interest. The aim of this preliminary study was to investigate parents'/caregivers' attitude toward COVID-19 vaccination of their child under 12 years of age and to analyze the characteristics associated to acceptance or refusal of this vaccination.

## Materials and Methods

### Questionnaire Structure

A closed-ended questionnaire was proposed to caregivers (coded either as “parent” or “other caregiver”) of children aged <12 years who accessed the pediatric emergency department or a pediatric outpatient clinic at three major hospitals in Milan, Italy, from 20 September to 17 October 2021. Caregivers were approached in the emergency department's waiting room or before the outpatient visit. In the emergency department setting, caregivers of children accessing with priority codes (imminent risk for life) were excluded. Caregivers who gave their consent to participate, were interviewed by a trained researcher.

For the caregivers, socio-demographic variables, information about COVID-19 vaccination status including reasons for not being yet vaccinated, history of COVID-19 also in family members and in friends/acquaintances were collected. For the child, demographic variables, type of hospital setting (emergency department vs. outpatient visit) and reasons for emergency department access were collected. Finally, the caregiver's intention to have the child vaccinated against COVID-19 and reasons for any refusal were investigated. Six possible reasons for both the caregiver's refusal to get vaccinated and/or to have the child vaccinated were investigated using the 4 Cs model of vaccine hesitancy (i.e., confidence, complacency, convenience, calculation) (14, 15). In particular, the reasons “I don't trust (mass media, vaccination campaign decision makers, pharmaceutical companies)”, “The vaccine is not that effective..” and “I am afraid of the vaccine side effects/adverse events” were traced back to the confidence dimension (14). The reason “I don't think it's necessary..” was related to the complacency dimension (14). The reason “Getting vaccinated/Having my child vaccinated would be too complicated/difficult/time-consuming” referred to the convenience component (14). Finally, the reason “I got informed and to date vaccination is not helpful for me/my child” referred to the calculation dimension (15). All data were anonymously collected.

### Statistical Analysis

Statistical analyses were conducted using R software, version 4.0.0. Mean and standard deviation for numerical variables and frequencies and proportions for categorical variables were calculated. Firstly, mosaic plots were produced to graphically explore any relationship between the caregiver's intention to have the child vaccinated and the categorical variables coding for: i) COVID-19 primary vaccination course completed or under completion [yes; no], ii) previous COVID-19 [yes; no], iii) previous COVID-19 in a family member [yes: disease; yes: death; no], iv) previous COVID-19 in a friend/acquaintance [yes: disease; yes: death; no], v) type of hospital setting [emergency department; outpatient visit]. By analyzing the contingency tables reporting the propensity to vaccinate the child as the response variable and the aforementioned variables as independent variables, odds ratios (OR) with their 95% confidence intervals were calculated. Fisher's exact test was performed to test the overall association between the intention to have the child vaccinated and the other categorical variables. Since a univariate correlation analysis does not account for the overall association structure, we conducted an initial exploratory multivariate analysis by means of a multiple correspondence analysis (MCA) to visually understand which conditions in the “multivariate” space were closest to the propensity to vaccinate the child and, therefore, associated. Finally, two regression models were considered: a logistic regression model and a generalized linear model with a log-link function. The latter was performed to obtain prevalence ratios (PR). For the construction of the logistic regression model, an initial model containing as predictors all the variables considered in the univariate analysis (defined as “full”) was considered. By modeling restricted cubic splines (RCS) at three nodes, the possible presence of non-linear effects was checked for age which was the only continuous variable. The ANOVA test did not find statistical evidence of the presence of non-linear effects. From the “full” model, the Akaike Information Criterion (AIC) was performed to obtain a simplified model in favor of its better interpretability. Considering the coefficients of the logistic regression model, ORs were calculated in logarithmic form with their 95% confidence intervals. Subsequently, a generalized linear model considering as predictors the same variables of the final logistic regression model was performed. From the calculated model coefficients, PRs were extracted with their 95% confidence intervals. A *p* < 0.05 was considered as significant.

## Results

A total of 612 consecutive caregivers were invited to complete the questionnaire. Eight caregivers did not accept to participate (response rate = 98.7%). Three questionnaires were excluded due to compiling errors and 601 questionnaires were finally retained and analyzed.

Table 1 summarizes the characteristics of enrolled caregivers and children, as well as the intention to have the child vaccinated.

**Table 1:** Characteristics of enrolled caregivers and their child.

***n***		**601**
**Caregiver**		
Type (*n*, %)	Parent	589 (98.0)
	Other caregiver	12 (2.0)
**Age (mean, SD)**		39.2 (7.65)
Sex (*n*, %)	Female	448 (74.5)
	Male	153 (25.5)
Nationality (*n*, %)	Italian	457 (76.0)
	Other	144 (24.0)
Level of education (*n*, %)	Bachelor's degree or superior	319 (53.1)
	Not a bachelor's degree	282 (46.9)
Employment status (*n*, %)	Employed	490 (81.5)
	Unemployed	111 (18.5)
COVID-19 vaccination status (*n*, %)	Vaccinated	547 (91.0)
	Not vaccinated	54 (9.0)
History of COVID-19 (*n*, %)	Yes	101 (16.8)
	No	500 (83.2)
History of COVID-19 in a family member (*n*, %)	Got sick	228 (37.9)
	Dead	19 (3.2)
	Neither of the two	354 (58.9)
History of COVID-19 in a friend/acquaintance (*n*, %)	Got sick	329 (54.7)
	Dead	92 (15.3)
	Neither of the two	180 (30.0)
**Child**		
Age (mean, SD)		4.84 (3.78)
Sex (*n*, %)	Female	243 (40.4)
	Male	358 (59.6)
Emergency department vs. Outpatient visit (*n*, %)	Emergency department	392 (65.2)
	Outpatient visit	209 (34.8)
**Intention to vaccinate the child (** ***n*** **, %)**	Yes	311 (51.7)
	No	138 (23.0)
	Unsure	152 (25.3)

*The mean age of participating caregivers was 39.2 years (SD 7.65). A total of 448 (74.5%) were female and 153 (25.5%) were male. 457 (76%) caregivers were Italian. The mean age for children was 4.8 years (SD 3.78). A total 358 (59.6%) were male and 243 (40.4%) were female*.

*A total of 311 (51.7%) caregivers declared their intention to vaccinate their child against COVID-19, 138 (23%) stated they would not have their child vaccinated and 152 (25.3%) declared to be unsure. Collapsing “Refuser” and “Unsure” responders, 290 (48.3%) caregivers were “Hesitant” toward COVID-19 vaccination for their child*.

Tables 2, 3 report the reasons for caregivers' refusal to get vaccinated and to have the child vaccinated, respectively.

**Table 2:** Caregivers' reasons for refusing to be vaccinated against COVID-19. As the caregiver could express a maximum of two reasons, the cumulative percentage overcomes 100%.

***n* (%)**	**54 (9.0)**
“I am afraid of the vaccine side effects/adverse effects” (*n*, %)	45 (83.3)
“I don't trust (mass media, vaccination campaign decision makers, pharmaceutical companies..)” (*n*, %)	13 (24.1)
“I got informed and to date vaccination is not helpful for me” (*n*, %)	9 (16.7)
“The vaccine is not that effective (I can still get the COVID-19, the vaccine is less effective against the Delta variant..)” (*n*, %)	3 (5.6)
“Getting vaccinated would be too complicated/difficult/time-consuming” (*n*,%)	1 (1.9)
“I don't think it's necessary (there's not much risk for me to get the COVID-19 and/or COVID-19 is not a severe disease)” (*n*, %)	0 (0.0)

**Table 3:** Caregivers' reasons for refusing the COVID-19 vaccination for their child. As the caregiver could express a maximum of two reasons, the cumulative percentage overcomes 100%.

***n* (%)**	**138 (23.0)**
“I am afraid of the vaccine side effects/adverse effects” (*n*, %)	109 (79.0)
“I don't think it's necessary (there's not much risk for the child to get the COVID-19 and/or COVID-19 is not a severe disease)” (*n*, %)	29 (21.0)
“I don't trust (mass media, vaccination campaign decision makers, pharmaceutical companies..)” (*n*, %)	27 (19.6)
“I got informed and to date vaccination is not helpful for my child” (*n*, %)	12 (8.7)
“The vaccine is not that effective (the child can still get the COVID-19, the vaccine is less effective against the Delta variant..)” (*n*, %)	7 (5.1)
“Having my child vaccinated would be too complicated/difficult/time-consuming” (*n*,%)	1 (0.7)

*A total of 547 (91%) caregivers completed or were about to complete the COVID-19 primary vaccination course, while 54 (9%) were not vaccinated. Among this latter group, the two main reasons for refusing vaccination were “I am afraid of the vaccine side effects/adverse effects” (83.3%) and “I don't trust (mass media, vaccination campaign decision makers, pharmaceutical companies..)” (24.1%). Interestingly, both reasons refer to the confidence dimension of the “4 Cs” model of vaccine hesitancy*.

*Among caregivers refusing to have their child vaccinated (N = 138, 23%), the two main reasons were “I am afraid of the vaccine side effects/adverse effects” (79.0%) and “I don't think it's necessary (there's not much risk for the child to get the COVID-19 and/or COVID-19 is not a severe disease)” (21%). A change in the vaccine hesitancy's dimensions occurred when caregivers were asked about the child vaccination, as the first reason refers again to the confidence dimension, whereas the second one refers to the complacency dimension*.

*Among caregivers refusing to get vaccinated (N = 54, 9%), 41 (76%) refused vaccination for their child, 7 (13%) declared to be unsure and 6 (11%) were in favor of their child's vaccination. On the other hand, among caregivers refusing vaccination for their child (N = 138, 23%), 41 (29.7%) were not vaccinated but 97 (70.3%) were vaccinated or were about to complete COVID-19 vaccination*.

In the univariate analysis, graphical exploration through mosaic plots showed a clear effect between the caregiver vaccinated or about to complete vaccination and the intention to vaccinate the child (data not shown). This association was also confirmed by the Fisher's exact test and an OR (with 95% confidence interval) of 10.05 (4.20, 29.3) was found. Hence, vaccinated caregivers show a 10-fold higher propensity to vaccinate their child compared to those not vaccinated. In the other cases, the Fisher's exact test failed to find any statistical evidence of an association between the propensity to vaccinate the child and the categories of the other variables.

**Figure 1:**
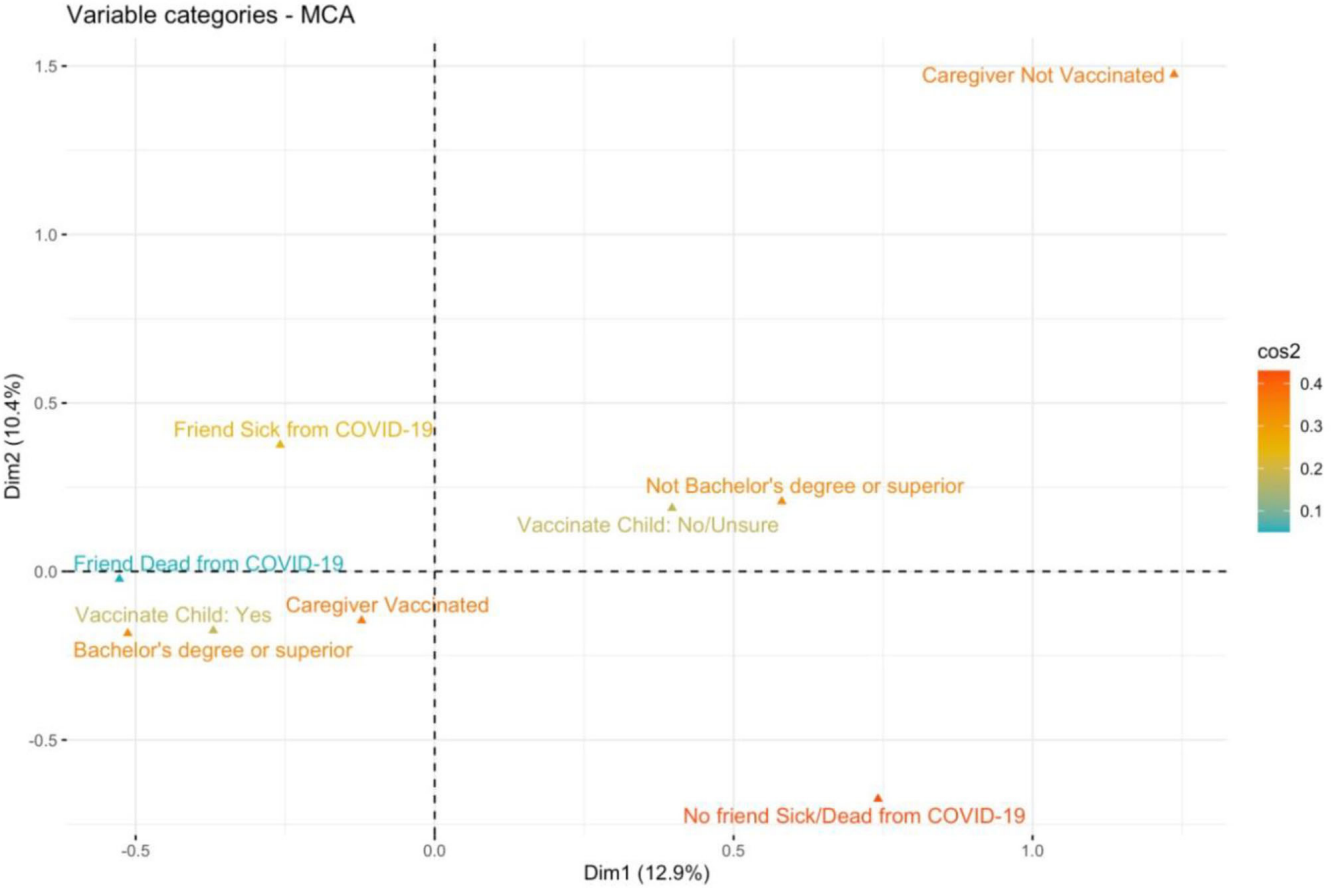
All levels of the categorical variables considered in the univariate analysis were projected in a new space defined by the first two dimensions (Dim1, Dim2) computed by MCA. Only variables showing a possible joint association to the caregiver's intention to vaccinate the child are displayed. The level “Vaccinate Child: Yes” (i.e., the caregiver willing to vaccinate the child), the level “Bachelor's degree or superior” (i.e., the caregiver holding a bachelor's degree or higher level of education) and the level “Caregiver Vaccinated” (i.e., the caregiver has completed/is about to complete the primary vaccination course) are close in the multivariate space, indicating a possible joint association. The categories of the variables are colored considering the parameter of cos2, which measures the degree of association between variable categories and a particular axis. If a variable category is well-represented by two dimensions, the sum of the cos2 is closed to one and tends to red color.

Figure 1 shows the results of the Multiple Correspondence Analysis. A caregiver's history of SARS-CoV-2 infection or having a family member who got ill or died due to COVID-19 were not associated to the intention to vaccinate the child.

**Figure 2:**
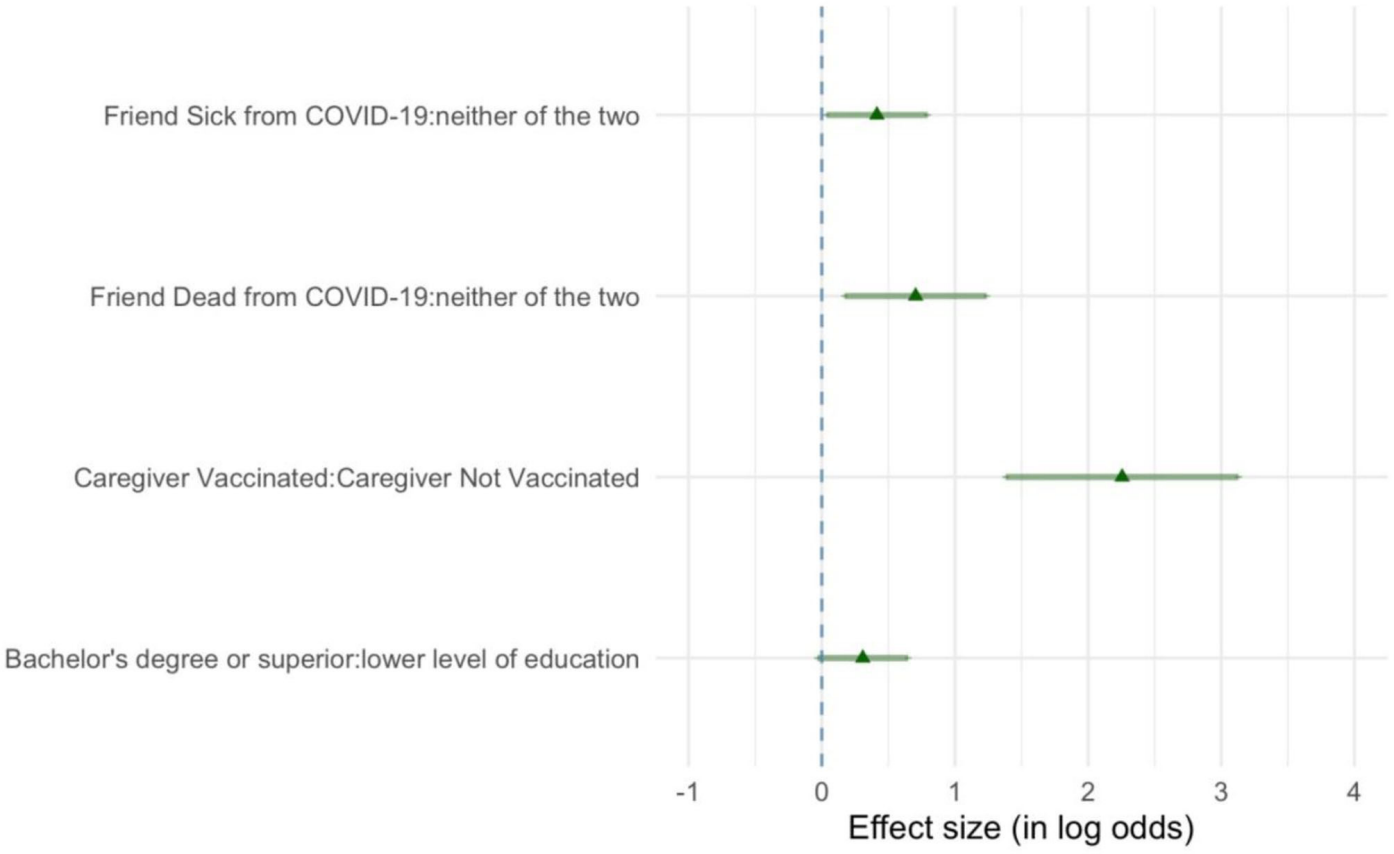
In this forest plot, the strength of association between the different conditions and the propensity to vaccinate the child are represented by means of the logarithmic form of the odds ratios computed by modeling a logistic regression model. Here, the conditions positively associated to the propensity to vaccinate the child are represented. The greater the logarithms of the odds ratios, the stronger is the association between a given condition and the propensity to vaccinate the child. Triangles indicate the point estimation while whiskers represent the 95% confidence intervals. For the caregiver having a friend or acquaintance who got ill or died due to COVID-19, the reference level is “neither of the two” conditions; for the caregiver's COVID-19 vaccination status, the reference level is “Caregiver Not Vaccinated”; for the caregiver's level of education, the reference level is “lower level of education.” Reference level means that when computing the odds ratio, the condition is put at the denominator.

In the logistic regression model, three variables of interest were identified: the vaccination against COVID-19 of the caregiver, having a bachelor's degree or higher level of education, and a history of a friend or acquaintance with SARS-CoV-2 infection (Figure 2).

The strongest predictor in favor of the child's vaccination was a caregiver who got vaccinated against COVID-19. The likelihood to vaccinate the child was greater also for caregivers with a bachelor's degree or higher level of education, compared to those having a lower degree of education. Finally, the intention to have their child vaccinated was higher among caregivers with a history of a friend/acquaintance who got ill due to COVID-19, and even grater if a friend/acquaintance died due to COVID-19.

**Figure 3:**
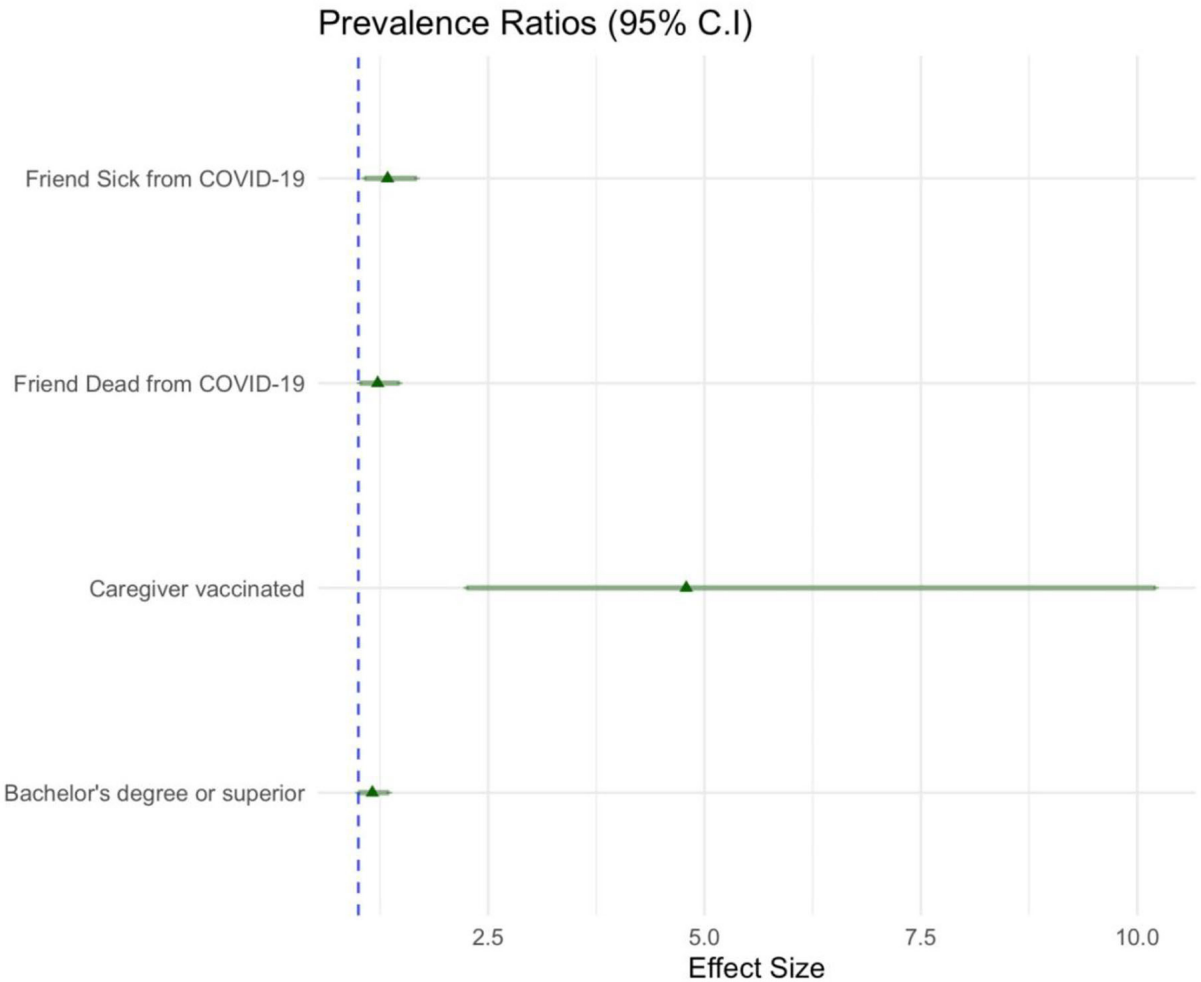
As in Figure 2, the strength of association between the conditions and the propensity to vaccinate the child are represented here by means of the prevalence ratios (PRs) indicated as triangles and whiskers (95% confidence intervals). Only the conditions positively associated to the propensity to vaccinate the child are displayed. The blue dashed line indicates a prevalence ratio equal to one (the condition displayed in the plot has an equal effect related to the condition with which is compared). Again, the condition “Caregiver vaccinated” is compared with its reference level “Caregiver Not Vaccinated”; the conditions “Friend Sick from COVID-19” and “Friend Dead from COVID-19” are compared with the reference level “neither of the two”; the condition “Bachelor's degree or superior,” referring to the caregiver's level of education, is compared with its reference level “lower level of education”.

In Figure 3, the forest plot shows the prevalence ratios (PRs) calculated by generalized linear models. The coefficients of the model resulted the same as those of the logistic regression model. A higher prevalence in the propensity to vaccinate the child was found for caregivers who were vaccinated, had a bachelor's degree or higher level of education, and had a history of a friend/acquaintance who became ill or died due to COVID-19.

## Discussion

To the best of our knowledge, this is the first study investigating parental vaccine hesitancy with a focus on children <12 years of age in Italy. The preliminary data provided by this multi-center survey shows that about half of the Italian caregivers are hesitant toward a possible COVID-19 vaccination for their child. On the other hand, caregivers vaccinated against COVID-19, those with high educational level, or with a history of friends/acquaintances who became ill or died due to COVID-19 were more willing to vaccinate their child.

Previous research addressed the caregivers' attitude toward COVID-19 vaccination for their children and conflicting findings were reported. A study conducted before the COVID-19 vaccine authorization in Europe observed that few caregivers (~10%) were hesitant about the pediatric vaccination against COVID-19 (27). A more recent study among Italian caregivers found that 26% of parents of adolescents between 12 and 17 years of age were in favor of COVID-19 vaccination (28). Considering the results of these studies and those of the present survey, a tendency toward an increasing acceptance of COVID-19 pediatric vaccination among caregivers might be speculated. On the other hand, a high percentage of caregivers is still hesitant toward COVID-19 vaccination. These findings confirm that vaccine hesitancy is more common in high-income countries as compared to low/middle-income ones (29). A previous survey investigating parental acceptance of COVID-19 vaccination in childhood found that the hesitancy was significantly higher in Russia (~70%) and U.S.A. (~35%) than in other low/middle-income countries in Asia, Africa and South America (~20%) (30). Similar results were found in a study conducted in Canada that observed 37% of hesitant parents (31).

This study also investigated factors possibly associated to the willingness to vaccinate young children. Three significant conditions emerged from our analysis, namely the caregiver attitude toward COVID-19 vaccination for him/herself, the educational level of the caregiver, and a history of a friend/acquaintance who got ill or died due to COVID-19. Interestingly, the first condition was the strongest predictor for parental intention to vaccinate their child. On the contrary, having a family member who got ill or died from COVID-19 was not significantly associated to parental intention to vaccinate their child. While in the clinical setting we often attribute parental vaccine hesitancy to the fact that in high-income countries, children are not affected anymore by potentially life-threatening diseases such as diphtheria or poliomyelitis, our study suggests that even significant experience of other people affected by COVID-19 might play a minor role in the attitude toward COVID-19 vaccination. This finding points out that understanding the caregiver's decision-making process also about the attitude toward his/her own vaccination, might provide new useful insights regarding vaccine acceptance or refusal for their children.

Although the history of friends ill or died due to COVID-19 is a non-modifiable factor, caregivers with a lower educational status might be considered as a potential target for tailored intervention to promote vaccination among children <12 years of age. It might be speculated that these caregivers are more vulnerable to misinformation and unproven theories on vaccines widely available on social media platforms (32). This result confirms that increasing efforts should be devoted to providing the whole population with evidence-based and easy-to-understand information on COVID-19 vaccination (33).

In this survey, two main reasons were provided about refusal of pediatric vaccination: (1) concerns about vaccine safety and (2) the assumption that COVID-19 is a mild condition in childhood. Both these assumptions are not supported by scientific evidence. A recently published trial showed that side effects following COVID-19 vaccination were mild in children aged 5 to 12 years and mainly included local reactions (7). On the contrary, the COVID-19 might present with life-threatening manifestations and long-term sequalae in this age group (34, 35).

COVID-19 has long been considered a disease mainly affecting adults (36–38). However, increasing data show that COVID-19 is becoming a pediatric illness: only 3% of COVID-19 cases were children at the beginning of the pandemic, compared to about 25% of cases nowadays (39). In young symptomatic subjects, COVID-19 might present with a wide spectrum of manifestations ranging from a lower tract respiratory infection (40) up to a multisystem inflammatory syndrome (41). In the U.S.A., approximately 700 fatal cases of COVID-19 occurred among children so far (39). These data point out that the decision not to get or to postpone COVID-19 vaccination is not without risks and caregivers should be aware about the possible effects of this choice. Moreover, focusing on COVID-19 vaccination for young children, also “indirect benefits” should be considered. Available data consistently report that school closures, impaired family well-being and parental stress due to the current pandemic are worldwide prevalent (42–44). Moreover, children and adolescents vaccination is considered essential to achieve a meaningful degree of community protection and eventually herd immunity (10, 11).

All these aspects emphasize the responsibility of healthcare providers and authorities to address increasing efforts in the promotion of pediatric vaccination.

This study has some limitations. Firstly, it was limited to three centers of Milan, Northern Italy. Future research should study the vaccine hesitancy among caregivers of children <12 years on a national basis and in other countries. Secondly, the interviews were conducted in a hospital setting. Indeed, it is possible that the concerns for the health status of the child might have an impact on the answers of the parents. However, previous studies showed that addressing vaccine hesitancy in this setting provide reliable results (45–47). All the questions of this survey were structured. Qualitative studies addressing the reasons underlying parental vaccine hesitancy for children <12 years might provide new helpful insights on this issue. We did not collect data on the income of the participants. Finally, we did not investigate the history of SARS-CoV-2 infection among children, which might modulate caregivers' disposition toward vaccination of their child (48).

## Conclusions

These preliminary results show that about half of caregivers of children <12 years of age in Italy are hesitant toward a COVID-19 vaccination for their child. The willingness to vaccinate their child was higher in caregivers vaccinated against COVID-19, in those with a high educational level, and in those with a history of friends/acquaintances who became ill or died due to COVID-19. Considering the availability and potential of COVID-19 vaccination for children <12 years of age (49), increasing efforts are necessary to provide evidence-based information to caregivers and to promote vaccination in this pediatric age group.

## Data Availability Statement

The raw data supporting the conclusions of this article will be made available by the authors, without undue reservation.

## Ethics Statement

Ethical review and approval was not required for the study on human participants in accordance with the local legislation and institutional requirements. The patients/participants provided their written informed consent to participate in this study.

## Author Contributions

ML and SC conceptulized the study. ML, GPM, GZ, GBa, PM, CA, and SC wrote the study design and gave a significant contribution to data interpretation. GBi performed the statistical analyses and contributed to data interpretation. ED'A, LC, CP, ES, and PT performed the data collection and contributed to data interpretation. ML, SC, GPM, and PM wrote the first draft of the manuscript. All authors reviewed the manuscript and approved the final verison as submitted.

## Conflict of Interest

The authors declare that the research was conducted in the absence of any commercial or financial relationships that could be construed as a potential conflict of interest.

## Publisher's Note

All claims expressed in this article are solely those of the authors and do not necessarily represent those of their affiliated organizations, or those of the publisher, the editors and the reviewers. Any product that may be evaluated in this article, or claim that may be made by its manufacturer, is not guaranteed or endorsed by the publisher.
